# Analysis of diagnostic test outcomes in a large loiasis cohort from an endemic region: Serological tests are often false negative in hyper-microfilaremic infections

**DOI:** 10.1371/journal.pntd.0012054

**Published:** 2024-03-14

**Authors:** Luzia Veletzky, Kirsten Alexandra Eberhardt, Jennifer Hergeth, Daniel Robert Stelzl, Rella Zoleko Manego, Ruth Kreuzmair, Gerrit Burger, Johannes Mischlinger, Matthew B. B. McCall, Ghyslain Mombo-Ngoma, Ayôla Akim Adegnika, Selidji Todagbe Agnandji, Pierre Blaise Matsiegui, Bertrand Lell, Peter Kremsner, Benjamin Mordmüller, Dennis Tappe, Michael Ramharter

**Affiliations:** 1 Department of Medicine I, Division of Infectious Diseases and Tropical Medicine, Medical University of Vienna, Vienna, Austria; 2 Department of Tropical Medicine, Bernhard Nocht Institute for Tropical Medicine & I. Dep. of Medicine, University Medical Center Hamburg-Eppendorf, Hamburg, Germany; 3 Centre de Recherches Médicales de Lambaréné, Lambaréné, Gabon; 4 German Center for Infection Research (DZIF), Hamburg-Lübeck-Borstel-Riems, Germany; 5 Department of Urology, University Medical Center Hamburg-Eppendorf, Hamburg, Germany; 6 Institute of Tropical Medicine, University of Tübingen, Tübingen, Germany & German Center for Infection Research, partner site Tübingen, Tübingen, Germany; 7 Department of Internal Medicine, Infectious Diseases, University Hospital Frankfurt, Goethe University, Frankfurt am Main, Germany; 8 Radboud University Medical Center, Department of Medical Microbiology, HB Nijmegen, The Netherlands; 9 Department of Implementation Research, Bernhard Nocht Institute for Tropical Medicine & I. Dep. of Medicine, University Medical Center Hamburg-Eppendorf, Hamburg, Germany; 10 Centre de Recherches Médicales de la Ngounié, Fougamou, Gabon; 11 National Reference Centre for Tropical Pathogens, Bernhard Nocht Institute for Tropical Medicine, Hamburg, Germany; National Institutes of Allergy and Infectious Diseases, NIH, UNITED STATES

## Abstract

**Background:**

The parasitic disease loiasis is associated with significant morbidity and mortality. Individuals with hyper-microfilaremia (greater than 20,000 microfilariae per mL of blood) may suffer from serious treatment-related or spontaneous adverse events. Diagnosing loiasis remains complex and primarily relies on direct parasite detection. In this study, we analyzed the performance of various diagnostic tests and the influence of parasitological and clinical factors on test outcomes in samples from individuals living in an endemic region.

**Methods:**

Data and samples were collected from rural Gabon. Loiasis was defined as either detectable microfilaremia, or a positive history of eyeworm as assessed by the RAPLOA questionnaire. Diagnostic testing included a quantitative PCR (qPCR) for detection of *Loa loa* DNA in blood samples, an in-house crude *L*. *loa* antigen IgG ELISA, and a rapid test for antibodies against the Ll-SXP-1 antigen (RDT). Sensitivity and specificity were determined for each test and factors potentially influencing outcomes were evaluated in an exploratory analysis.

**Results:**

ELISA, RDT and qPCR results were available for 99.8%, 78.5%, and 100% of the 1,232 participants, respectively. The ELISA and RDT had only modest diagnostic accuracy. qPCR was specific for *L*. *loa* microfilaremia and Cycle threshold values correlated with microfilarial density. Anti-*L*. *loa* IgG levels were highest in occult loiasis, and antibody levels correlated inversely with *L*. *loa* microfilarial density as did RDT line intensities. Only 84.6% and 16.7% of hyper-microfilaremic individuals tested positive by ELISA (11/13) and RDT (2/12), respectively.

**Conclusion:**

None of the tests demonstrated high sensitivity and specificity for loiasis. Indirect diagnostic assays were characterized by low specificity. Additionally, hyper-microfilaremic individuals often tested negative by RDT and ELISA, indicating that these tests are not suitable for individual case management in endemic populations.

## Author summary

Loiasis remains highly neglected despite recent evidence of significant loiasis-associated mortality and morbidity. This complex disease is difficult to diagnose, especially in endemic populations. Here, we assessed the performance of several diagnostic assays. In addition, an exploratory analysis of factors associated with the outcomes of indirect, serology-based tests was performed. The presented data show only moderate performance of indirect tests. Furthermore, amicrofilaremic loiasis was associated with higher quantitative serological test results. None of the indirect tests detected all cases of microfilaremic loiasis, including individuals with high and hyper-microfilaremia. As a negative test may not reliably exclude hyper-microfilaremic infections, these serological tests should not be used as screening tests in individual case management. This is important, as hyper-microfilaremic individuals are at increased risk of developing serious adverse events after treatment with drugs such as ivermectin or diethylcarbamazine.

## Introduction

The parasitic disease loiasis is endemic in Central Africa and parts of West Africa. In comparison to river blindness and lymphatic filariasis, loiasis and its associated clinical manifestations and pathophysiology are not as well studied [1]. However, health care professionals working on loiasis have repeatedly advocated for loiasis-centered research. This call to action is supported by recent estimates indicating significant morbidity and mortality directly attributable to loiasis [2–6]. A better understanding of the disease, as well as development of adequate diagnostics and appropriate treatment leading to improved patient care is needed [2–5,7–12]. Importantly, a proportion of patients harbor extremely high loads of asexual larval stages in their peripheral blood, exceeding 20,000 microfilariae/mL. This hyper-microfilaremic state is associated with higher risk of spontaneous and treatment-related severe clinical manifestations [3,13].

Microfilaremia diagnostics in individual patients still primarily rely on direct parasite detection using microscopy, showing similar sensitivity as polymerase chain reaction (PCR) [14]. However, the parasite is also known to cause “occult” loiasis, where patients harbor adult filariae, but no detectable microfilariae. This group of patients represents a large proportion of infected individuals in endemic regions [9,15–17]. Currently, occult loiasis can only be diagnosed based on symptoms reported by the patient, indicating the presence of adult filaria, such as subconjunctival migration (eyeworm). While this sign is considered reasonably specific for the disease, it does not occur in all infected individuals. There is, therefore,–at least in theory–a group of patients without a history of eyeworm migration and without peripheral microfilaremia, that current diagnostic tests cannot reliably identify. Importantly, the use of serological assays is limited in endemic regions, as they do not allow differentiation between active and past infections and are limited by a high level of cross-reactivity with other helminth infections. Furthermore, there are no biomarkers identified to date for the reliable detection of amicrofilaremic loiasis [18–22]. Recently, a rapid diagnostic test for antibodies against the recombinant *L*. *loa* SXP-1 antigen (Ll-SXP-1-RDT) has been developed and has been proposed as an epidemiological screening tool for loiasis [23]. Laboratory-based assessments of the diagnostic accuracy of Ll-SXP-1-RDT using samples positive for *L*. *loa* and other filarial diseases have shown promising results with good sensitivity and specificity [23,24]. However, test performance data from endemic regions are still limited [25]. Thus, an analysis of the diagnostic accuracy of various diagnostic tests was performed. An exploratory analysis of the influence of various factors, including demographics, parasitic co-infections, as well as subgroups of loiasis on test outcomes was conducted. Analyzed samples were collected from individuals living in a loiasis endemic region. Assessed tests included the lateral flow Ll-SXP-1-RDT, an in-house IgG enzyme-linked immunosorbent assay (ELISA) and a quantitative PCR (qPCR) for detection of *L*. *loa* DNA in blood.

## Methods

### Ethics statement

Ethical approval for the study was provided by the institutional ethics committee (Comité d’Ethique Institutionnel du Centre de Recherches Médicales de Lambaréné; IORG0007336/IRB00008812; CEI-011/2017). Prior to all study-related procedures, written informed consent was obtained from each participant or their legal representative.

### Sample and data collection

**Fig 1:**
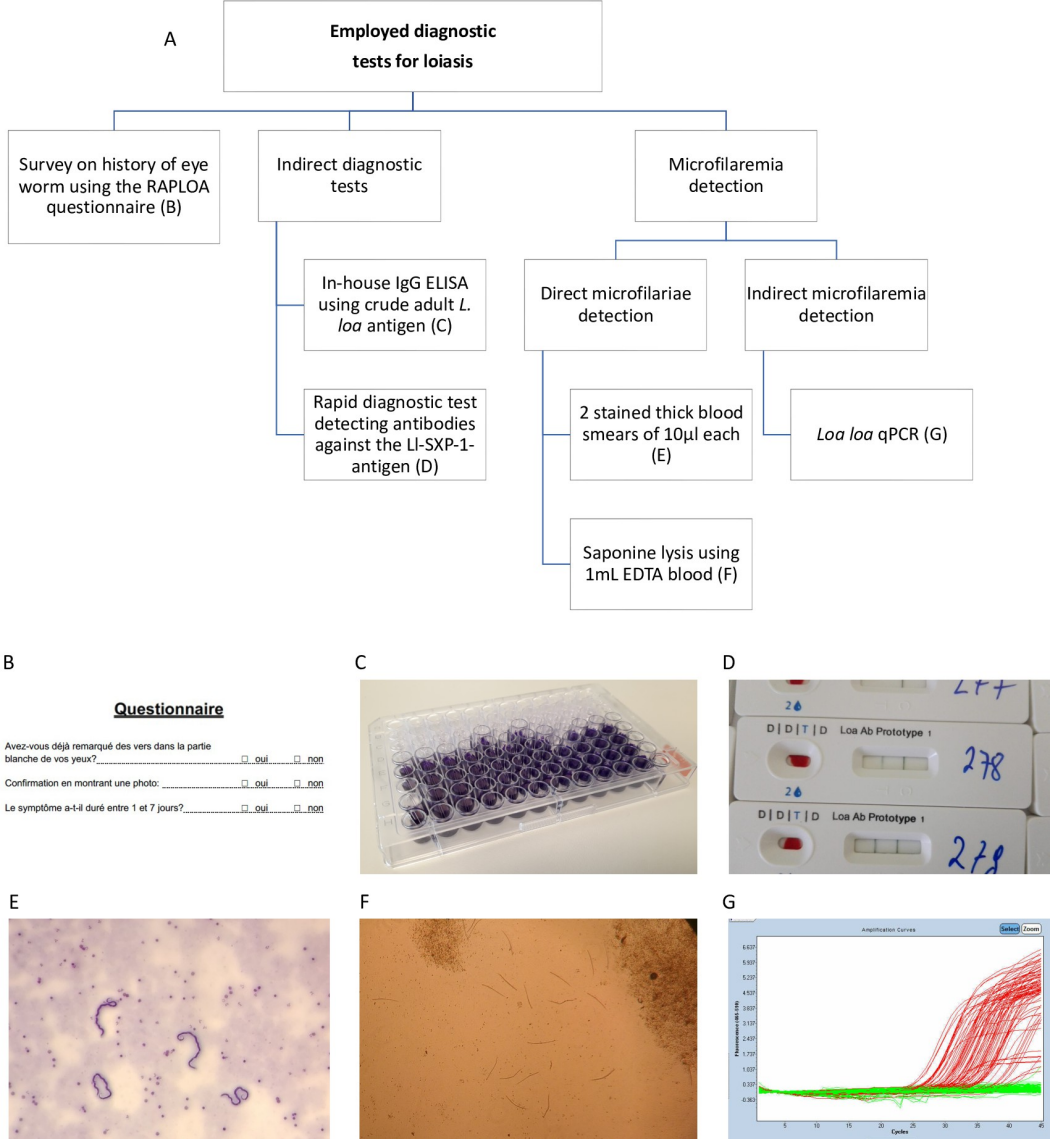
Overview of employed diagnostic procedures. (A) Diagnostic scheme. Procedures included history of eyeworm (B) assessed by the RAPLOA questionnaire, an in-house *Loa loa* IgG ELISA using crude adult worm antigen (C), a rapid diagnostic test detecting antibodies against the Ll-SXP-1 antigen (D), direct microfilaria detection including microscopy of Giemsa-stained thick blood smears (E) and saponin lysis (F), as well as a *L*. *loa* qPCR (G).

Samples and data used in this analysis were collected during a cross-sectional survey conducted in a high *L*. *loa* transmission region that has previously been described in detail [4,26]. In brief, in 2017 and 2018 a community survey was performed in central Gabon, aiming to assess the burden of disease associated with loiasis. Venous blood samples were collected between 10am and 3pm in tubes containing ethylene-diamine-tetra acetic acid (EDTA). The survey used a standardized questionnaire to capture baseline data of participants and occurrence of loiasis-related symptoms. History of eyeworm during the previous year and over participants’ lifetimes was assessed using the standardized “Rapid Assessment Procedure for Loiasis” (RAPLOA) questionnaire [27]. Fig 1 provides an overview of all applied diagnostic methods.

### Analysis of blood samples

Each blood sample was handled according to a standardized processing scheme to ensure uniform pre-analytic and analytic conditions. On the day of collection, samples were aliquoted for immediate and future procedures. Immediate procedures were carried out within three days of sample collection and included detection of *L*. *loa* and *Mansonella spp*. microfilariae by microscopy, as well as performing the Ll-SXP-1-RDT. Thick blood smears were simultaneously read according to the Lambaréné method for detection of *Plasmodium spp*. This was done since both *Mansonella* and *Plasmodium* spp. are frequent parasitic co-infections in the study region [28–30]. If Plasmodia were detected, malaria treatment was provided according to national guidelines. Subsequently, an in-house *L*. *loa* IgG ELISA and a qPCR detecting *L*. *loa* DNA, as well as a PCR assay for the detection of *Mansonella perstans* and *sp*. *deux* were done. EDTA blood and plasma aliquots were immediately frozen at -20°C, transferred within a week to -80°C for long-term storage and later shipped for further analysis to Hamburg, Germany.

### Microscopic microfilaria detection

Direct detection of microfilaria was achieved by light microscopy of two 10 μl Giemsa-stained thick blood smears. As microscopy using 20μl blood smears has a detection limit of about 50mf/mL, a second diagnostic step was performed for negative blood samples to increase diagnostic sensitivity. This concentration technique used 1mL EDTA blood and added saponin for blood lysis, followed by centrifugation and subsequent microscopy of the leukocyte pellet (adapted from Bouyou Akotet et al., 2016) [31]. The full laboratory protocol is provided in the appendix file S1 Text. Microfilaremia is reported as microfilariae per mL (mf/mL).

### Loa loa and Mansonella microfilaria PCR

DNA was extracted from 400μl of EDTA blood using the AltoStar Purification Kit 1.5 and the AltoStar Automation System AM16 (Altona Diagnostics GmbH, Mörkenstr. 12, 22767 Hamburg, Germany) following manufacturer instructions. The PCR was adapted from a previously published method by Fink et al.[14], only the quencher was changed to BHQ1. All PCR parameters are provided in Table 1. qPCR results were defined as positive if at least two out of three of the triplicates per sample had a Cycle threshold (Ct) value below 41. Results were categorized as positive vs. negative based on absolute Ct values. To detect co-infection with *Mansonella perstans* and *Mansonella sp*. *deux*, a real-time PCR Fluorescence Resonance Energy Transfer (FRET) assay, targeting an internal transcribed spacer (ITS) region present in both Mansonella types, was designed. Species specificity of the assay was confirmed by a melting curve analysis, sequencing of PCR products and comparison of sequences in the Basic Local Alignment Search Tool (BLAST). The assay was designed using the LightCycler Probe Design Software 2.0 by Roche with the Design Type Mutation HypProbe. PCR settings are provided in Table 1.

**Table 1:** The PCR settings for the *Loa loa* qPCR and *Mansonella* real-time FRET assay.

***Loa loa* qPCR**	
The targeted amplicon of the PCR has a length of 62bp. The PCR was run on a LightCycler480 under the following conditions:	
Primer	LL-MF72-F 5’-CGGAAGACTCAACGTCAGAAATCA- 3’
	LL-MF72-R 5’- AGGAACGCTTGATGGTGATGT- 3’
	LL-MF72-P 5’- FAM- CCAACAGCCTGCTTTT-BHQ1- 3’
Mastermix	Luna Universal Probe qPCR MasterMix
PCR settings	95°C—60 sec; (95°C 15 sec, 60°C 45 sec) x 45 cycles, 37°C 30 sec
	For the assay, a non-template negative control consisting of water and a positive control using confirmed *Loa loa* DNA was used. Additionally, a *L*. *loa-*negative but *Mansonella spp*.-positive sample was used as a second negative control. All tests were run in triplicate.
**FRET assay for detection of *Mansonella perstans* and *Mansonella sp*. *deux***	
The targeted amplicons of the PCR have a length of 181bp for the *Mansonella species*. Species discrimination was based on the presence of a single nucleotide polymorphism, reducing the affinity of the probe, and leading to different melting temperatures for *Mansonella perstans* (60.2°C) and *Mansonella sp*. *deux* (56.1°C).	
Primer	Mans-FRET-ITS-F 5’-CCTAAACCGTCGATAATGATGA-3’
	Mans-FRET-ITS-R 5’-CACCGCTAAGAGTTAAAAATTTC-3’
	Mans-FRET-ITS-S Cy5’-AATACACACATACATATACTAATTGTAATTATTGA-3’ Phosphat
	Mans-FRET-ITS-A 5’-AATAAGCATTTATGCTAAATATGCTACCAACAAAT-3’ 6-FAM
Mastermix	MgCl2, Solution S, Puffer BD, dNTPs, a HotStart Taq Polymerase (Solis)
PCR settings	95°C 15min; (95°C 20sec, 60°C 45sec, 72°C 20sec) x 45cycles; 72°C 5min
Melting curve analysis	Temperature range: 35°C to 75°C
	For the assay, a non-template negative control consisting of water and a positive control using confirmed *Mansonella perstans* and *sp*. *deux* DNA was used. Additionally, a *Mansonella spp*. *-*negative but *L*. *loa*-positive sample was used as a second negative control. All tests were run in triplicate.

### In-house crude Loa loa antigen IgG ELISA

Samples were analyzed using an in-house crude *L*. *loa* antigen IgG ELISA. For coating of microtiter wells, an aqueous extract of sonicated adult *L*. *loa* from an infected individual in Central Africa was used as antigen at a dilution of 1:6000 in skimmed milk powder/phosphate-buffered saline. Secondary alkaline phosphatase (AP)-conjugated goat anti-human IgG antibodies (dilution of 1:10,000, Jackson ImmunoResearch Europe, Ely, United Kingdom) and p-nitrophenyl phosphate were added (Sigma-Adrich/Merck, Taufkirchen, Germany), and the reaction was quantified using an ELISA reader. Sera from 30 healthy Caucasian blood donors were used in the validation process of this test to determine the cut-off threshold for positivity (15 arbitrary units) by adding 3 times the standard deviation to the arithmetic mean of test results from healthy donors. Using this cut-off in individuals with no exposure to nematodes, the ELISA showed 100% specificity. However, using a total of 41 archived sera (exact numbers per disease in parenthesis) from patients with a definitive diagnosis of mansonellosis (5), dirofilariosis (4), onchocercosis (5) Brugia malayi infection (1), Wuchereria infection (4), dracunculosis (2), ascariasis (4), strongyloidiasis (4), schistosomiasis (4), cystic echinococcosis (4) and malaria (4), resulted in cross reactions, leading to an overall specificity of 44% in the validation process. Sensitivity of the ELISA for loiasis was 89% based on 18 archived sera samples from patients with diagnosed loiasis (presence of adult worm or detectable microfilaremia). These sera were not from patients in the current loiasis cohort.

### Ll-SXP-1-RDT

The Ll-SXP-1-RDT produced by Drugs & Diagnostics for Tropical Diseases (San Diego, CA, USA, Lot Number 67–0002) was performed under field conditions in the first 967 participants of the study within three days following blood sampling. The RDT is designed to detect IgG against the *Loa loa-SXP-1* antigen. As described by the manufacturer’s guide, 5μL of EDTA blood were placed in the designated mold using a calibrated micropipette, followed by 2 drops of eluent. After 15 minutes, the test cassette was put into the dedicated smartphone reader device which performed an automated measurement of line intensity. The intensity of reader units (RU) was provided as absolute numbers. Positive and negative controls for the RDT were performed regularly as recommended by the manufacturer. The RDT was considered positive if the smartphone reader detected an intensity of the test line of 600 RU or higher and an intensity of the control line of 400 RU or higher. This cutoff was chosen based on previously published results, indicating a high sensitivity and good specificity of this cutoff [23].

### Definition of loiasis

Loiasis positivity was defined as either a positive life-time history of eyeworm detected by the RAPLOA questionnaire and/or microscopically detectable microfilaremia. Individuals with neither were defined as loiasis-negative. Loiasis-positives were further divided into two subgroups: microfilaremic loiasis with microscopically detectable *L*. *loa* microfilaremia, and occult loiasis with a positive history of eyeworm but no microscopically detectable microfilaremia. Thus, the two subgroups were mutually exclusive. To assess the influence of the extent of microfilaremia on test outcomes, microfilaremic individuals were further divided into low (1–7,999 mf/mL), high (8,000–19,999 mf/mL) and hyper-microfilaremic (≥20,000 mf/mL) loiasis. These cutoffs are employed for the risk assessment of spontaneous or treatment-related adverse events [1,26]. A sensitivity analysis using a modified case definition including a positive eyeworm history during the previous year and/or microfilaremia was done to assess possible influence of the case definition.

### Data management

Data were collected on paper-based forms and were manually entered into a database (Microsoft Access). Statistical analysis was performed using the software package R (Version 4.0.5, R Foundation for Statistical Computing, Vienna, Austria) and STATA/BE 17.0 (StataCorp, USA). Continuous variables were expressed as median (including interquartile range, IQR) and compared using the Wilcoxon-rank sum test. Categorical variables were compared using the χ2 test. Multiple logistic and linear regression models were applied to adjust for possible confounders, as deemed appropriate, and results were presented as odds ratio (OR) or coefficient (coeff.), respectively. Agreement between outcomes of binary test results was assessed by calculating Cohen’s κ. The Spearman’s rank correlation coefficient ρ was calculated as a measure of the strength of the relationship between continuous variables. Diagnostic test performance was evaluated using the R package ’caret’. Two-sided p-values are provided, and an α of 0.05 was determined as the cut-off for statistical significance.

## Results

### Study population

Samples from 1,232 participants were included in this study. A detailed description of the study population has been previously published [4,26]. In summary, 626 (50.8%) individuals were defined as loiasis-positive, of which 298 (24.2%) were found to be *L*. *loa* microfilaremic and 328 (26.6%) had occult loiasis. *Mansonella spp*. PCR was positive for 460 participants and *Plasmodium spp*. were found in thick blood smears of 287 participants (37.3% and 23.3%, respectively). Availability of samples for the respective diagnostic methods is provided below.

### Analysis of test performance for loiasis detection

qPCR results were available for all 1,232 participants and 20.1% (248/1,232) were *L*. *loa* qPCR positive. ELISA results were available for 1,229 (99.8%) of all participants and showed overall 90.2% (1,108/1,229) seropositivity. The RDT was done for the first 967 individuals included in the study. Results of 6 individuals were excluded as the control band was below 400 RU, resulting in the analysis of 78.0% (961/1,232) of participants. Overall, RDT-positivity was 41.3% (398/961). Sensitivity, specificity and positive as well as negative predictive values (PPV/NPV) are provided in Table 2. The sensitivity analysis yielded similar sensitivity and specificity results for the respective diagnostic assays. Results are provided in the appendix file S1 Table.

**Table 2:** Sensitivity, specificity, positive and negative predictive values of the *Loa loa* qPCR, in-house ELISA and Ll-SXP-1-RDT to detect loiasis defined by either a positive life-time history of eyeworm assessed by the RAPLOA questionnaire and/or detectable microfilaremia.

		N	n positive (row %)	n negative (row%)	% Sensitivity (95% CI)	% Specificity (95% CI)	% PPV (95% CI)	% NPV (95% CI)
***L*. *loa* microfilaria PCR**								
**Loiasis positivity**	**Pos.**	626	247 (39.5)	379 (60.5)	39.5 (36.7–42.2)	99.8 (99.6–100.0)	99.6 (99.2–100.0)	61.5 (58.8–64.2)
	**Neg.**	606	1 (0.2)	605 (99.8)				
**IgG ELISA against crude *L*. *loa* antigen**								
**Loiasis positivity**	**Pos.**	625	596 (95.4)	29 (4.6)	95.4 (94.2–96.5)	15.2 (13.2–17.2)	53.8 (51.0–56.6)	76.0 (73.7–78.4)
	**Neg.**	604	512 (84.8)	92 (15.2)				
**Ll-SXP-1 rapid diagnostic test**								
**Loiasis positivity**	**Pos.**	523	256 (49.0)	267 (51.0)	49.0 (45.8–52.1)	67.6 (64.6–70.5)	64.3 (61.3–67.4)	52.6 (49.4–55.7)
	**Neg.**	438	142 (32.4)	296 (67.6)				

### Exploratory analysis of the potential influence of various host and parasite factors on test outcomes

#### PCR

**Fig 2:**
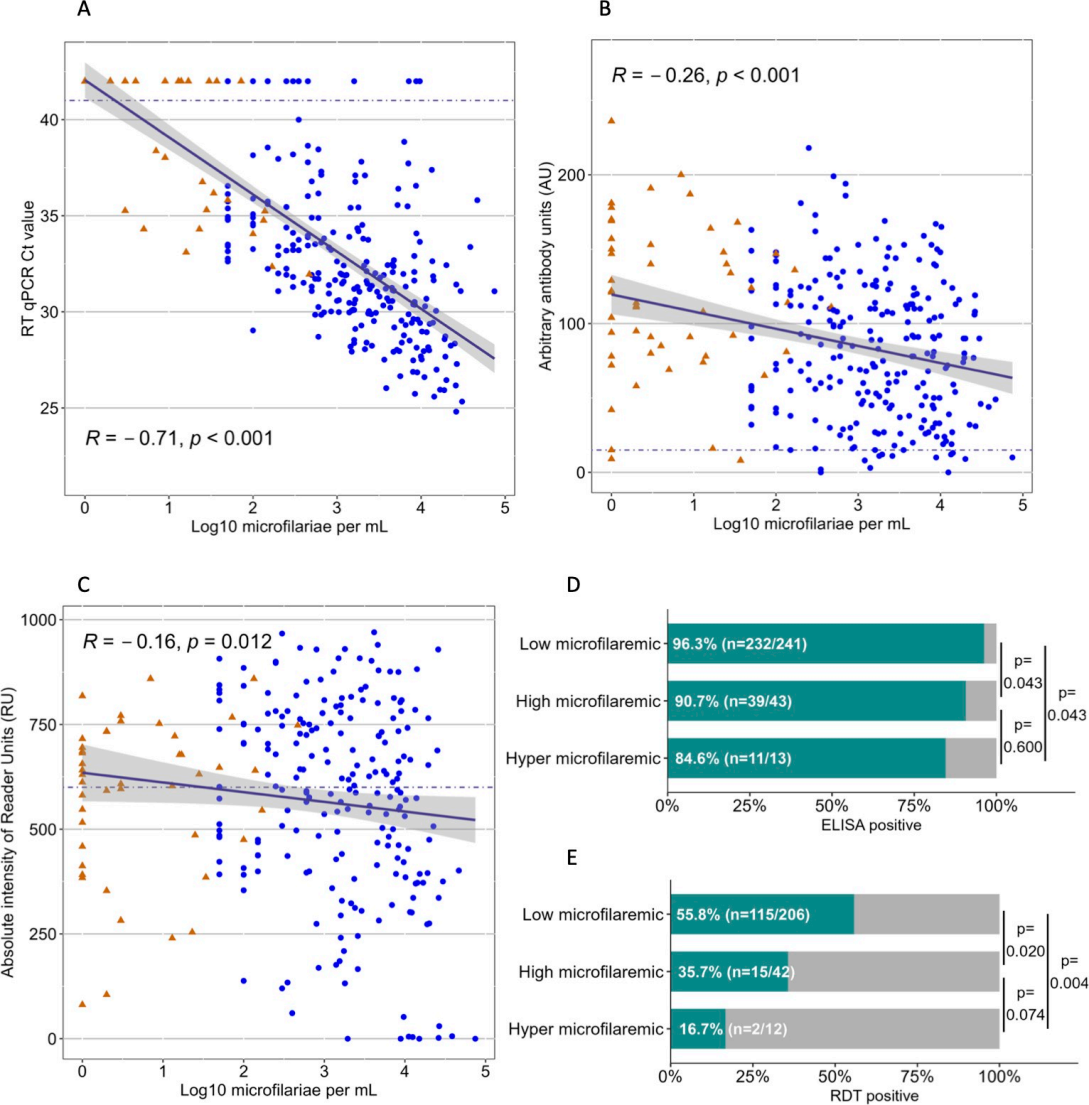
Results of exploratory test analysis of microfilaremic loiasis. Linear regression models showing the association between A) Ct values of the qPCR, B) arbitrary antibody units (AU) of the IgG ELISA and C) reader units (RU) of the Ll-SXP-1-RDT, and *L*. *loa* microfilarial density assessed by microscopy. Percentages of test positivity are shown for microfilaremic subgroups in IgG ELISA (D) and the Ll-SXP-1-RDT (E). In A to D, blue dots represent microscopy results of thick blood smears and orange triangles represent results from leukocyte concentration. In Fig 2A, the negative qPCR Ct values were displayed as a value of 42, which is outside of the detection area. Cut-off values for test-positivity are marked by dashed lines.

An analysis of the mean qPCR Ct value and the log-transformed *L*. *loa* microfilaremia per mL of all microfilaremic individuals showed an inverse association (ρ = -0.71, p <0.001), Fig 2A. In total, 57 individuals were microscopically microfilaremic but qPCR negative. Of those, 52 (91.2%) had a microfilaremia below 1,000 mf/mL. Of 6 samples that were qPCR positive but microfilaria-negative via microscopy (0.5%, 6/1,232), 5 had a positive history of eyeworm.

#### In-house crude L. loa antigen IgG ELISA

Analysis of the in-house ELISA by age showed that 76.9% (120/156) of individuals in the age group <15 years were seropositive. Seropositivity was similar in the adult age-groups (92.1% in 15 to 59-year-olds and 91.2% aged 60 years or older). There was no significant difference in seropositivity between female and male participants (χ2 = 0.159). Seropositivity was associated with *Mansonella* positivity (χ2 < 0.001, OR 9.70 (95% CI: 4.69–20.06), OR adjusted for loiasis and age group 8.32 (95% CI: 4.00–17.30)), but not with *Plasmodium* positivity (χ2 = 0.157). Thus, serological analyses were adjusted for age group and *Mansonella* status, but not for sex and *Plasmodium* positivity. Fig 2D and Table 3A present ELISA results. Loiasis-positives were more often seropositive than loiasis-negatives (χ2<0.001, OR 3.69 (2.39–5.70), age group and *Mansonella* adj.-p <0.001, aOR 2.58 (1.63–4.09)) and loiasis-positive subgroups had similar percentages of seropositivity. Seropositivity percentages in microfilaremic groups significantly decreased with increasing microfilarial density and differences are shown by post-hoc analyses (Fig 2D).

**Table 3:** A) An overview of ELISA seropositivity and arbitrary units (AU) as well as B) Ll-SXP-1-RDT positivity and absolute intensity of Reader Units (RU) by loiasis subgroups. *Arbitrary Antibody Units, ** Interquartile range, *** Wilcoxon-rank sum test comparing median AU or RU. ‡Participants were defined by microfilaremia density as low (1–7,999 mf/mL), high (8,000–19,999 mf/mL) and hyper-microfilaremic (≥20,000 mf/mL).

**A**							
**Loiasis status**			**N**	**n positive (row %)**	**χ2**	Median AU* (IQR**)	p-value***
**Negative**			604	512 (84.8)	<0.001	71 (29–118)	<0.001
**Positive**			625	596 (95.4)		96 (54–139)	
	**Occult**		328	314 (95.7)	0.642	105 (58–151)	<0.001
	**Microfilaremic**		297	282 (95.0)		90 (48–124)	
**Density of microfilaremia‡**							
		**Low microfilaremic**	241	232 (96.3)	0.005	91 (55–125)	0.008
		**High microfilaremic**	43	39 (90.7)		72 (31–105)	
		**Hyper- microfilaremic**	13	11 (84.6)		49 (32–90)	
**B**							
**Loiasis status**			**N**	**n positive (row %)**	**χ2**	Median RU* (IQR**)	p -value***
**Negative**			438	142 (32.4)	< 0.001	441 (199–641)	<0.001
**Positive**			523	256 (49.0)		593 (400–743)	
	**Occult**		263	124 (47.2)	0.408	585 (394–738)	0.373
	**Microfilaremic**		260	132 (50.8)		603 (419–747)	
**Density of microfilaremia‡**							
		**Low microfilaremic**	206	115 (55.8)	<0.001	630 (471–753)	0.001
		**High microfilaremic**	42	15 (35.7)		551 (372–665)	
		**Hyper- microfilaremic**	12	2 (16.7)		356 (24–483)	

Median ELISA arbitrary units (AU) in the overall study population were 84 (IQR: 39–131), and there were no significant differences by age group or sex. As shown in Table 3A, median AU were higher in loiasis-positives than in loiasis- negatives, and higher in occult loiasis compared to microfilaremic individuals. Loiasis-positivity and the level of AU were associated and remained so after correction for age group and *Mansonella*-positivity (p<0.001, coeff. 14.26 (7.84–20.68)). In the occult loiasis group where eye worm was seen during the previous year, median AU were 118 (IQR:64–158), compared to 98 (IQR: 52–140) if eye worm had been seen more than a year ago (p = 0.027). Individuals with reported Calabar swelling had a median AU of 104 (IQR: 58–146). In individuals where the Calabar swelling had occurred during the previous year, median AU were 108.5 (IQR: 61–147), and if it had been more than a year ago median AU were 78.5 (IQR: 39–129), (p = 0.009). In the group where eyeworm and Calabar swelling had occurred during the previous year, median AU were 117 (IQR: 68–152). AU correlated negatively with the presence of *L*. *loa* microfilaremia, and the density of microfilaremia (Fig 2B, ρ = -0.26, p = 0.001). As shown in Table 3A, higher peripheral microfilaremia was associated with lower median AU.

As serological negativity by ELISA was a rather rare finding, we specifically analyzed this subgroup. Overall, 121 of 1,229 individuals were found to be seronegative (9.9%). While seronegativity was similar between men and women (χ2 = 0.155; 8.5% and 10.9%, respectively), younger age was associated with seronegativity (χ2 < 0.001). Twenty-nine (24.0%) of the 121 seronegative individuals were loiasis-positive, of whom 14 (48.3%) had occult loiasis and 15 (51.7%) were microfilaremic. In the fifteen *L*. *loa* microfilaremic but seronegative individuals, median microfilaremia was 1,950 mf/mL (mean 10,206 mf/mL, min–max: 1–74,600 mf/mL), with 11 individuals harboring more than 1,000 mf/mL. Seronegative but *Mansonella-*positive individuals comprised 6.6% (8/121) of the study population. *Plasmodium*-positivity and seronegativity was found in 18.2% (22/121). Of the seronegatives, 91 individuals (75.2%) were neither loiasis- nor *Mansonella*-positive, and 7 (5.8%) were loiasis and *Mansonella*-positive.

#### Ll-SXP-1-RDT

RDT-positivity was similar in all age groups (33.8%, 41.8% and 44.5%, in individuals aged <15 years, 15–59 years and 60 years or older, respectively, χ2 = 0.122) and there was no sex difference (χ2 = 0.551). RDT-positivity was weakly associated with *Mansonella*-positivity (χ2 = 0.053), but not with *Plasmodium* spp. positivity (χ2 = 0.827). Therefore, RDT analysis was not further adjusted for any confounding factors. Fig 2E and Table 3B show RDT results. RDT-positivity was associated with loiasis-positivity (χ2 < 0.001, OR 2.00 (1.54–2.60)) and loiasis subgroups showed similar RDT-positivity percentages (χ2 = 0.408). RDT-positivity percentages significantly decreased with increasing microfilarial density (χ2 <0.001) and there were significant differences between microfilaremic groups (Fig 2E). Overall, the median RDT RU intensity was 536 U/L (IQR: 300–705). The median RU intensity was lowest in the youngest age group (<15 years of age) with 282 RU (IQR: 17–639), and higher in 15 to 59-year-olds with 540.5 (IQR: 319–699) and 575 RU (IQR: 396.5–734) in individuals aged 60 years or older (overall p<0.001). Female participants had a median RU of 524 (IQR: 295–698) and males had a median RU of 556 (IQR: 232–717), (p = 0.244). Median RU were higher in loiasis-positives than in loiasis-negatives, irrespective of *Mansonella*-positivity (p-adj < 0.001, coeff. 131.27 (99.62–162.9)). Microfilaremic and occult loiasis individuals had similar median RU intensities. In the occult loiasis group where eye worm was seen during the previous year, the median RU were 614.5 (IQR: 436–774), if the eyeworm was seen more than a year ago the RU were 545 (IQR: 332–711), (p = 0.007). Individuals with reported Calabar swelling had a median RU of 573 (IQR: 384–712), and the time of occurrence was not found to be associated with the level of RU. The level of RDT RU negatively correlated with density of microfilaremia (Fig 2C, Spearman’s ρ = -0.16, p = 0.012). Analysis of median RU by microfilaremia group showed that higher peripheral microfilaremia was associated with a lower median RU (Table 3B).

## Discussion

We assessed the performance of different diagnostic methods for *L*. *loa* infections and performed an exploratory analysis of various host and parasite factors and their effects on test outcome in a large population in an endemic area. None of the evaluated tests showed a satisfactory performance. While the overall analysis revealed an insufficient accuracy of serologic tests, limitations of these results, possibly influenced by the choice of the case definition, need to be discussed. One of the main limitations in test analysis is the missing gold standard for loiasis diagnosis. We set the case definition and reference standard for test performance analysis as a positive history of eyeworm based on the standardized RAPLOA questionnaire and/or microscopically detectable microfilaremia [27]. It is important to note that direct detection of microfilaremia is inherently limited in sensitivity by the amount of examined blood. We chose a two-step diagnostic approach. Thick blood smears followed by a concentration technique using 1 mL of blood were used to raise diagnostic sensitivity of microscopy. Yet some individuals may still have been misclassified as amicrofilaremic. Importantly, excess mortality and morbidity have been associated with any detectable *L*. *loa* microfilaremia [3,6,13,32]. Thus, identification of all microfilaremic patients is an important objective of loiasis diagnostics.

On the other hand, occult loiasis must not be ignored. It has been shown that individuals with occult disease have a high disease burden but are often overlooked and misdiagnosed, due to inherent limitations of blood-based diagnosis [9,26].

Furthermore, distinguishing active from past infection in patients with occult loiasis is difficult and can currently only be estimated based on time since last eyeworm occurrence or other signs of migrating adult filariae. To assess the possible impact of the case definition, a sensitivity analysis including only individuals with eyeworm occurrence during the previous year was done, revealing similar results. However, possible active infection without occurrence of eyeworm is hard to prove or refute. This subgroup may contribute to the high proportion of positive serological tests in individuals who were defined as negative.

One of the assessed diagnostic methods was a qPCR on blood samples for the detection of peripheral microfilaremia. The qPCR provided similar results as microscopy, adding little benefit [14,18]. However, one of the aims of developing a qPCR for loiasis was to be able to detect the density of microfilaremia with a higher throughput than microscopy, an important factor especially in the context of larger control programs. The assessed qPCR has previously been described as having a good correlation with microfilaremia in laboratory-based settings [14]. This was also found in this large sample set collected under field conditions. Importantly, sensitivity for the detection of microfilaremia was lower with qPCR in comparison to microscopic methods, especially in samples with microfilaremia <1,000 mf/mL. This is most likely due to the volume of blood used for DNA extraction, which was less than the final volume used in microscopy. Furthermore, it is important to note that DNA extraction from *L*. *loa* microfilariae is technically challenging due to their thick sheath. Thus, results may have been compromised by the DNA extraction method used.

Exploratory analysis of demographic and clinical factors associated with serology-based tests revealed important findings. Seropositivity by the IgG ELISA was very high and there were no sex differences, but positivity was associated with age. Overall, 76.9% in the under 15-year-old age group were positive and serological negativity was rare. Both findings are in line with previous reports [20–22]. Interestingly, comparison of different loiasis sub-groups showed lower median AU in the microfilaremic population than in patients with occult loiasis. Similar findings have previously been described [8,31,33,34]. Of note, highest AU were observed in individuals who had reported an eyeworm migration and Calabar swelling within the previous year, both signs caused by migrating adult filariae. Analysis by density of microfilaremia revealed that high and hyper-microfilaremic individuals were more likely to be seronegative compared to low microfilaremic patients. Furthermore, the level of AU was found to inversely correlate with the density of microfilaremia. Comparison with and interpretation of available serological results in literature is difficult, due to the use of different antigens, assay protocols and definitions of loiasis [18–21]. However, an association between IgG subclass levels and loiasis infection types, i.e. microfilaremic or occult loiasis, has repeatedly been described [19,20]. Further, it was found that Loa-specific IgG4 positivity can reach up to 100% in individuals living in high transmission areas [21]. Here, only total IgG was analyzed rather than specific subclasses, but still differences between loiasis subgroups were found. Interestingly, the assay was surprisingly insensitive for the detection of hyper-microfilaremic cases. Additionally, a strong serological cross-reactivity with *Mansonella*-positivity was seen. However, *L*. *loa* microfilaremia was associated with lower antibody levels while *Mansonella* microfilaremia was not. Thus, the underlying effect may be specific to *L*. *loa* microfilaremia.

The second indirect test assessed was a RDT detecting antibodies against the Ll-SXP-1-antigen. This RDT was developed to map *L*. *loa* prevalence in epidemiological surveys, including mass drug administration programs [23]. The RDT has been assessed under laboratory conditions and showed promising results. Sensitivity and specificity for loiasis-detection was also high, when tested against samples positive for other filarial infections [23,24]. In one study, the RDT was employed in the field, but individual diagnostic features of the test were not described [25]. Although RDT results were not available for our entire cohort, the analysis presented here is the largest and most comprehensive evaluation of this RDT in an endemic setting, with more than 960 study participants tested. Importantly, results of the RDT were collected using an automated smart phone reader, which allowed exact quantification of band intensity, referred to as RU. While band intensity has previously been shown to correlate well with antibody values, it should be noted that these values may not follow a linear correlation [23]. Based on the case definition used in the current study, sensitivity and specificity were low. Further, the RDT was cross reactive with *Mansonella* spp. microfilaremia. Comparing *L*. *loa* microfilaremic subgroups, we saw that low microfilaremic individuals had the highest RU intensities. Interestingly, the higher the microfilaremia, the lower the RU. As with the ELISA, the RDT was often false-negative in highly microfilaremic individuals. Indeed, this effect was even stronger for the RDT. Of the 10 hyper-microfilaremic samples which were classified as negative, 4 samples would have also been negative if the RDT had been read by the naked eye as they showed a RU below 100, which is the reported visibility-cutoff [23]. It needs to be emphasized that in this subgroup there is less potential for misclassification, as high microfilaria counts can be incontestably diagnosed even in small amounts of blood. This contradiction to previous results may be explained by the different study settings, populations, definitions of loiasis, prevalence of co-infections and the use of samples with lower microfilaremia [23,24]. The high proportion of false negativity of ELISA and RDT in hyper-microfilaremic individuals is of particular concern, as, based on these findings, a negative serological test cannot reliably exclude hyper-microfilaremia.

The frequent negativity of hyper-microfilaremic samples in both serological assays may be explained by a type of prozone effect. High levels of microfilaria in the EDTA blood samples could have blocked the antibody reaction mechanically or by direct binding of the antibodies. Another possible mechanism, particularly for the negative correlation between microfilarial density and quantitative test results, may be the absence or reduced number of specific (protective) antibodies, which facilitates the presence of microfilaremia. This could be due to an inability to mount an effective immune response by the host or by direct suppression of the immune response by the parasite.

Thus, additional analyses, such as measuring levels of IgG subclasses, would be important to better understand these findings. The possible prozone effect may be further investigated by testing serial dilutions of EDTA blood and plasma. It should be kept in mind that the RDTs were conducted using fresh EDTA blood and retesting of frozen and thawed samples may yield different results. Nevertheless, further testing of the samples needs to be performed to shed more light on these results.

Importantly, the findings were adjusted for some parasitic co-infections, including malaria and *Mansonella spp*. However, results for other co-infections with possible immunomodulatory effects, such as soil-transmitted helminths or schistosomiasis, were not available and were thus not considered in the statistical analysis.

## Conclusion

This analysis showed insufficient test accuracy of diagnostic tests for loiasis in an endemic population. Importantly, we found that individuals with high microfilarial loads frequently had false negative serological results by IgG ELISA and Ll-SXP-1-RDT. While further studies are needed to understand underlying mechanisms, these diagnostic methods should not be used as a basis for treatment initiation in individual case management with potentially harmful drugs such as ivermectin or diethylcarbamazine.

## Supporting information

S1 Text Saponin lysis laboratory protocol. (DOCX)

S1 Table Sensitivity analysis with a loiasis case definition of eyeworm history during the previous year and/or detectable microfilaremia. (DOCX)

S1 Database Database of the study. (XLS)
