# Supplementary material for: Analysis of diagnostic test outcomes in a large loiasis cohort from an endemic region: Serological tests are often false negative in hyper-microfilaremic infections
Source: PLoS Negl Trop Dis. 2024 Mar 14;18(3):e0012054. doi: 10.1371/journal.pntd.0012054 (PMC10965051; doi:10.1371/journal.pntd.0012054)
Supplement: S1 Text — (DOCX) [file pntd.0012054.s001.docx]

Saponin lysis laboratory protocol

*Procedure*

Saponin- saline solution preparation (1% in Saponine in NaCl)

5g of Saponin

500ml 0.9% NaCl

1. Mix the saponin gently with the NaCl in a 50 ml Falcon Tube
2. Prepare freshly every week
3. Store at 2-8°C

Saponin Lysis steps

1. add 1ml of EDTA blood to a 15ml falcon tube and add an equal amount of saponin-solution.

2. Mix gently and leave for 5 minutes until all RBC are lysed.

3. Centrifuge hemolysate at RT for 10 mins at around 2.000RPM.

4. discard the supernatant and transfer the sediment onto a slide.

6. examine entire preparation for microfilaria using the 10x and 40x magnification

7. count all microfilaria in the preparation

8. sample can be stained if necessary for species identification.
