# Supplementary material for: Analysis of diagnostic test outcomes in a large loiasis cohort from an endemic region: Serological tests are often false negative in hyper-microfilaremic infections
Source: PLoS Negl Trop Dis. 2024 Mar 14;18(3):e0012054. doi: 10.1371/journal.pntd.0012054 (PMC10965051; doi:10.1371/journal.pntd.0012054)
Supplement: S1 Table — (DOCX) [file pntd.0012054.s002.docx]

Sensitivity analysis with a Loiasis case definition of eyeworm history during the previous year and/or detectable microfilaremia.

|  |  | **N** | **n positive (row %)** | **n negative (row%)** | **% Sensitivity (95% CI)** | **% Specificity (95% CI)** | **% PPV (95% CI)** | **% NPV (95% CI)** |
| --- | --- | --- | --- | --- | --- | --- | --- | --- |
| ***L. loa* microfilaria PCR** | | | | | | | | |
| **Loiasis with recent eyeworm** | **Pos.** | 460 | 245 (53.3) | 215 (46.7) | 53.3 (48.6- 57.9) | 99.6 (98.9-99.9) | 98.8 (96.5-99.7) | 78.1 (75.4-80.7) |
|  | **Neg.** | 771 | 3 (0.4) | 768 (99.6) |  |  |  |  |
| **IgG ELISA against crude *L. loa* antigen** | | | | | | | | |
| **Loiasis with recent eyeworm** | **Pos.** | 459 | 441 (96.1) | 18 (3.9) | 96.1 (93.9-97.7) | 13.4 (11.1-16.0) | 39.8 (36.9-42.8) | 851 (77.5-90.9) |
|  | **Neg.** | 769 | 666 (86.6) | 103 (13.4) |  |  |  |  |
| **Ll-SXP-1 rapid diagnostic test** | | | | | | | | |
| **Loiasis with recent eyeworm** | **Pos.** | 388 | 199 (51.3) | 189 (48.7) | 51.3 (46.2-56.4) | 65.3 (61.2-69.2) | 50.0 (45.0-55.0) | 66.4 (62.4-70.3) |
|  | **Neg.** | 573 | 199 (34.7) | 374 (65.3) |  |  |  |  |
